# Dental Health in Children with Congenital Heart Defects: A Systematic Review and Meta-Analysis

**DOI:** 10.3390/jcm13237022

**Published:** 2024-11-21

**Authors:** Carol Moussa, Guillaume Savard, Laurent Estrade, Rim Bourgi, Naji Kharouf, Frédéric Denis, Maha H. Daou

**Affiliations:** 1 Faculty of Dentistry, University of Tours, 37032 Tours, France; guillaume.savard@univ-tours.fr (G.S.); frederic.denis@univ-tours.fr (F.D.); maha.daou@univ-tours.fr (M.H.D.); 2 Division of Education, Ethics, Health, Faculty of Medicine, University of Tours, 37044 Tours, France; 3 Department of Medicine and Bucco-Dental Surgery, Tours University Hospital, 37044 Tours, France; l.estrade@chu-tours.fr; 4 Department of Restorative Dentistry, School of Dentistry, Saint-Joseph University, Beirut 1107 2180, Lebanon; rim.bourgi@net.usj.edu.lb; 5 Department of Biomaterials and Bioengineering, INSERM UMR_S 1121, University of Strasbourg, 67000 Strasbourg, France; dentistenajikharouf@gmail.com; 6 Department of Endodontics and Conservative Dentistry, Faculty of Dental Medicine, University of Strasbourg, 67000 Strasbourg, France; 7 Department of Pediatric Dentistry, Faculty of Dental Medicine, Saint Joseph University, Beirut 1107 2180, Lebanon; 8 Craniofacial Research Laboratory, Division of Biomaterials, Saint Joseph University, Beirut 1107 2180, Lebanon

**Keywords:** congenital heart defects, dental caries, DMF index

## Abstract

**Background:** Oral health outcomes in children with Congenital Heart Defects (CHD) have significant implications. The aim of this systematic review and meta-analysis is to update the current understanding of oral health outcomes in children with CHD and compare caries prevalence between CHD children and healthy controls. **Methods:** All studies between 2014 and 2024 comparing oral health status between children with and without CHD were considered for inclusion. Studies had to use the DMF/dmf index (Decayed, Missing, Filled Teeth or Surface index), in permanent and deciduous teeth. Two separate meta-analyses were conducted: one analyzing DMFS scores and another focusing on dmft scores. Medline, Central, and Embase databases were screened. Twelve articles were included in the qualitative synthesis, and two studies were finally included in each quantitative synthesis. **Results:** Several studies identified significant differences in oral health outcomes, suggesting that children with CHD are at a higher risk of dental caries compared with healthy controls, particularly as they become older. However, the differences were not uniformly observed across all studies and age groups. Based on the meta-analysis, there was no statistically significant difference in either DMFS scores (MD: 0.07 [95% CI: −0.13, 0.27]; *p* = 0.48) or in dmft scores (MD: 1.39 [95% CI: −1.05, 3.83]; *p* = 0.26). **Conclusions:** This systematic review and meta-analysis highlight a possible increased risk of dental caries in children with CHD, although results were not statistically significant and varied across studies. More standardized and rigorous studies are required to provide clearer insights into oral health outcomes for this population.

## 1. Introduction

Children with congenital heart defects (CHD) present a vulnerable population with an increased risk for dental caries and periodontal issues [1], which, if left untreated, can lead to severe complications such as infective endocarditis [2,3]. Several studies have shown the existence of a two-way interaction between oral pathogens and systemic conditions in CHD patients. On one hand, CHD patients often present with an altered oral microbiome, which shifts toward more pathogenic species due to immune dysregulation, frequent medical interventions, and compromised oral hygiene. Pathogens like *Streptococcus mutans* and *Porphyromonas gingivalis* are prevalent and contribute to oral diseases such as dental caries and periodontitis, with the potential to enter the bloodstream and trigger systemic inflammation, further aggravating cardiovascular conditions [4]. On the other hand, the systemic condition itself—characterized by immune compromise and frequent medical treatments—creates an environment that favors the growth of these harmful oral bacteria, as seen in CHD children with early childhood caries, who show elevated levels of cariogenic and periodontal pathogens like *Streptococcus sobrinus* and *Prevotella* [5]. Additionally, oral inflammation caused by periodontal pathogens, such as *Porphyromonas gingivalis* and *Treponema denticola*, can escalate systemic inflammation and contribute to vascular complications by promoting chronic inflammatory responses [6]. This two-way relationship highlights the reciprocal influence between oral and systemic health, making the maintenance of both essential for improving the overall quality of life in CHD patients. Any disruption in this balance can negatively affect patients’ quality of life [7], underscoring the importance of maintaining both oral and cardiac health.

Children with CHD have shown a higher prevalence of dental caries due to several factors, including medication, sociodemographic characteristics, tooth brushing habits, dental history, and comorbidities [8]. However, and although several studies have explored the oral health outcomes in these children, comprehensive and up-to-date reviews of the literature are limited. Notably, the most recent reviews have included studies published between 2000 and 2019. Previous research has primarily relied on systematic reviews without the inclusion of meta-analytic techniques, limiting the ability to draw concrete, data-driven conclusions about the extent of caries risk in children with CHD [9].

This study focuses specifically on caries risk rather than periodontal status in order to quantify the impact of oral health using a single, standardized metric: the DMFT score (Decayed, Missing, and Filled Teeth). To explore the prevalence of dental caries, standardized indices are used and recommended by the World Health Association (WHO). According to WHO, the DMFT score is the sum of the number of Decayed, missing due to caries, and Filled Teeth in the permanent teeth. The mean number of DMFT is the sum of individual DMFT values divided by the sum of the population [10]. The dmft/DMFT (dmfs/DMFS) indices are essential tools in dental epidemiology used to assess the prevalence and severity of dental caries. DMFT applies to permanent dentition, while dmft is for primary dentition. The DMFS and dmfs indices similarly assess decayed, missing, and filled surfaces in permanent and primary teeth. These indices provide concrete, quantifiable numbers that represent the overall caries burden in a population, making them especially useful for comparing different groups. Given that children with CHD are often more vulnerable to oral health issues, the dmft/DMFT indices are a practical and reliable way to quantify their oral health status [11].

Therefore, the aim of this systematic review and meta-analysis is to update the current understanding of oral health outcomes in children with CHD and compare caries prevalence between CHD children and healthy controls.

## 2. Materials and Methods

### 2.1. Data Sources

This study adheres to the Cochrane Guidelines and Preferred Reporting Items for Systematic Reviews and Meta-Analyses (PRISMA) [12], with its protocol registered with INPLASY under the identifier INPLASY2024100074. A review protocol was established prior to the study, with the PICO model defined as follows: Is there a difference in the oral health status (O) between children under 18 years old (P) with congenital heart defects (I) compared to healthy children (C)?

### 2.2. Search Strategy

The literature search was completed on 28 July 2024. Two of the authors (C.M. and M.H.D.) independently conducted the search across the bibliographic databases of Medline, Embase, and Central. Separate searches were performed using search strings (Table 1).

### 2.3. Selection Criteria

C.M. and M.H.D. conducted the article selection process by independently assessing the titles and abstracts of the articles. The inclusion criteria required studies to compare oral health status between children with and without CHD and to use the DMF/dmf index (Decayed, Missing, Filled Teeth or Surface index) for permanent and deciduous teeth. In this review, we prioritized studies using the DMF index due to its prevalence in the literature and its utility in assessing dental health in children with congenital heart defects. Future research incorporating the ICDAS II index could provide additional insights by capturing early and nuanced stages of caries development [8]. As filters, the search was limited to studies published between 2014 and 2024 and to articles available in English. Letters to the editor/authors, commentaries, expert opinions, reviews, conference abstracts, case reports, case series, and pilot studies were all excluded. Articles that passed the initial screening proceeded to full-text analysis for confirmation of final inclusion.

### 2.4. Data Extraction and Analysis

All studies that met the inclusion criteria were identified and reviewed, with any disagreements resolved through consensus before data extraction from each selected study.

For the meta-analysis, a software program, Review manager v5.4.1 (Cochrane Collaboration, Oxford, UK) was used to estimate the odds ratio (ORs) with 95% confidence intervals (CIs). Continuous data were analyzed using the Mantel–Haenszel Chi2 test and presented as weighted mean difference (WMD) with 95% CIs. A *p* value < 0.05 was considered significant. Heterogeneity was assessed using a Chi2 test on N−1 degrees of freedom, with significance defined at an alpha risk of 0.05 and the I2 test was applied to quantify heterogeneity [13]. Pooled estimates were calculated using a random effects model to account for variability between studies. Funnel plot analysis was used to assess potential publication bias for each outcome. All statistical analyses were performed using Review manager v5.4.1 (Cochrane Collaboration).

### 2.5. Assessing the Risk of Bias

**Figure 1:**
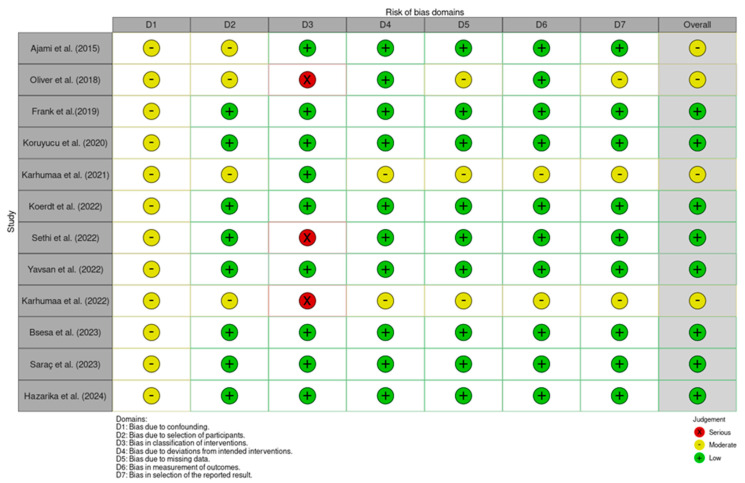
ROBINS-I assessment for the studies [7,14,15,16,17,18,19,20,21,22,23,24].

Bias in the included studies was assessed using the ROBINS-I (Risk Of Bias In Non-randomized Studies of Interventions) tool (Figure 1), which is suitable for evaluating non-randomized and observational studies. This tool examines seven domains of bias: confounding, selection of participants, classification of interventions, deviations from intended interventions, missing data, measurement of outcomes, and selection of the reported result. Confounding bias was considered moderate when important factors, such as socioeconomic status or oral hygiene practices, were not controlled for. Selection bias was marked as moderate for retrospective studies that relied on pre-existing records, which could introduce non-random selection. Classification bias was typically low since CHD and control groups were clearly defined. Missing data bias was assessed as moderate in studies using retrospective data, where incomplete records were possible. Measurement bias was usually low due to the use of standardized caries indices (DMFT/dmfs), while reporting bias was deemed low when all outcomes were reported without selective omissions.

## 3. Results

### 3.1. Selected Articles

**Figure 2:**
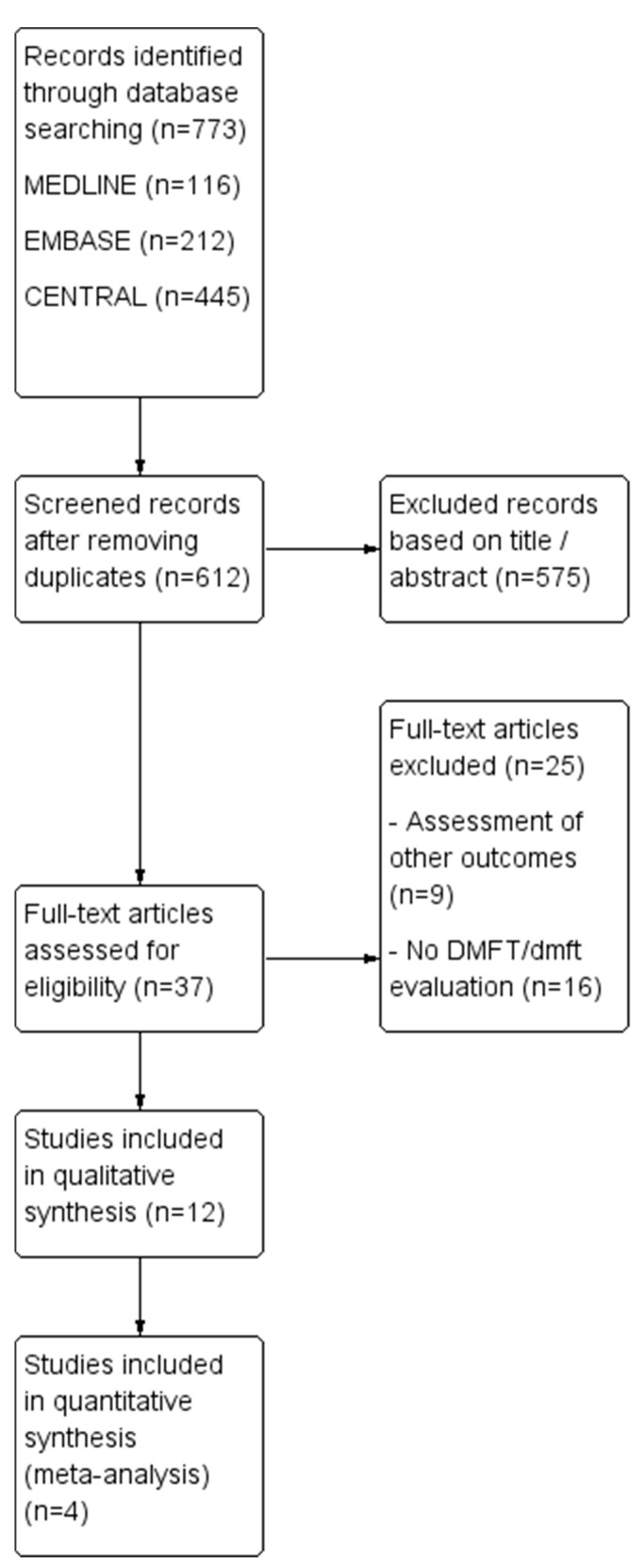
Study flow diagram of inclusion process for articles selected.

A total of 773 potential publications were initially identified through the database search. After removing duplicates, 612 articles remained for title and abstract screening, leading to the exclusion of 575 articles. Thirty-seven articles were then deemed eligible for full-text review, of which 12 met the criteria for inclusion in qualitative synthesis and 4 for meta-analysis (Figure 2).

### 3.2. Characteristics of the Included Studies for Qualitative Synthesis

The characteristics of the included studies, comparing oral health outcomes between children with CHD and those without CHD, are summarized in Table 2. The extracted data included year of publication, authors, article title, study type, age range of the population, sex distribution, number of participants in each group, mean values (±standard deviation) of the studied indices, and *p*-values where available.

### 3.3. Statistical Findings in Selected Articles

Based on the findings from the included studies, several articles reported statistically significant differences between the two groups.

Statistical results were extracted from selected studies that compared oral health indices between children with congenital heart disease (CHD) and control groups. These studies reported significant or non-significant differences in various caries indices, such as DMFT, dmft, and DMFS, highlighting disparities in oral health outcomes between the CHD and control populations, as well as disparities in the results across the different articles (Table 3). Even though some articles provided statistical results, they were not included in this table due to differences in study design or analytical approach, such as comparing additional groups or using different classification systems.

### 3.4. Articles Selected for Meta-Analysis

Two distinct meta-analyses were performed. Regarding the comparison between the DMFS index in children with CHD and healthy controls, a fixed-effects model was used for analysis, as there was no significant heterogeneity between the studies (I^2^ = 0%).

**Figure 3:**
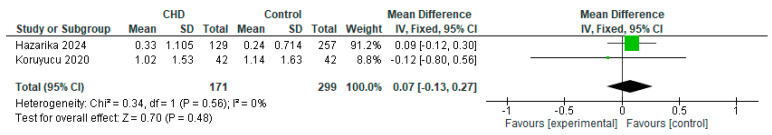
Forest plot of comparison of DMFS between children with CHD and healthy controls [17,18].

There was no statistically significant difference in DMFS scores between children with CHD and controls (MD: 0.07 [95% CI: −0.13, 0.27]; *p* = 0.48). This suggests that CHD does not significantly impact DMFS outcomes when compared to healthy controls based on the available data from the included studies (Figure 3).

Regarding the comparison of dmft scores between children with CHD and healthy controls, a random-effects model was used due to the high degree of heterogeneity between the studies (I^2^ = 92%). The meta-analysis showed no statistically significant difference in dmft scores between children with CHD and controls (MD: 1.39 [95% CI: −1.05, 3.83]; *p* = 0.26). While the Bsesa et al. [14] study reported a significant mean difference favoring the control group, the Yavsan et al. [7] study did not show a significant difference, contributing to the overall heterogeneity.

**Figure 4:**
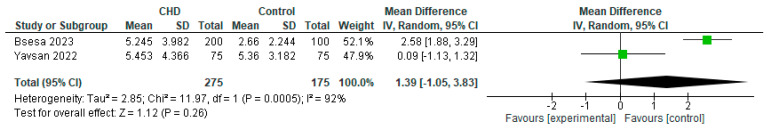
Forest plot of the comparison of dmft between children with CHD and healthy controls [7,14].

This suggests that, despite some variation in the findings, there is no conclusive evidence from the pooled results that children with CHD have higher dmft scores compared to controls (Figure 4).

## 4. Discussion

This systematic review and meta-analysis evaluated the oral health outcomes of children with congenital heart disease (CHD). With the systematic review we observed that the majority of studies reported statistically significant differences in dental caries prevalence between the two groups, with children with CHD generally experiencing worse oral health outcomes. For instance, Koerdt et al. found significant differences in DMFT/dmft scores in the 7–12 and 13–17 age groups, with children with CHD having worse scores, though the difference was not significant in younger children [15]. Similarly, Karhumaa et al. and Bsesa et al. found significantly higher DT, DMFT, and dmft indices in children with CHD at various age points, indicating a greater caries burden in this population [14,16]. Hazarika et al. also reported a significant difference in dmfs scores, though not for DMFS [17].

However, some studies, such as those by Yavsan et al. and Koruyucu et al., did not find statistically significant differences in caries indices between groups [7,18]. Notably, Koruyucu et al. also examined salivary profiles, finding that children with CHD had lower salivary pH and buffering capacity, as well as elevated oxidative stress markers, suggesting a broader systemic influence on their oral health [18].

Across the studies, a common finding was that children with CHD tend to experience worse oral health, specifically in terms of dental caries prevalence, as evidenced by elevated DMFT/dmft indices across multiple studies. Despite the variability in study designs and the use of different indices, this outcome was frequent throughout the review. These findings highlight the need for standardized oral health assessments in CHD populations [11].

Only four studies met the criteria for inclusion in the meta-analysis due to the need for comparable study designs, population characteristics, and index measurements.

The first meta-analysis, comparing DMFS indices, revealed no significant difference between children with CHD and controls, with the overall mean difference being non-significant. This finding suggests that, under similar conditions, DMFS scores may not capture a substantial disparity in oral health between CHD and non-CHD populations.

Similarly, the second meta-analysis, which focused on the dmft index, showed no significant difference between children with CHD and controls. However, this result was accompanied by high heterogeneity that can suggest that additional factors, such as variations in population characteristics or study methodologies, may have influenced the results, highlighting the need for further investigation into the causes of variability across studies. The observed heterogeneity could also be attributed to the difference in age ranges between the studied groups. Yavsan et al. [7] focused on children aged 3 to 6 years, while Bsesa et al. [14] included a broader range of children aged 4 to 12 years. Yavsan et al. found no significant difference between CHD and control groups, whereas Bsesa et al. reported a significant disparity, which may be explained by the increased risk of dental caries as children grow older, a conclusion supported by the findings in this systematic review. Given that the age group in Yavsan falls within the broader age group of Bsesa, this meta-analysis was attempted to gain insights from the limited number of studies eligible for quantitative synthesis. Despite the inherent bias in conducting this meta-analysis with such heterogeneity, it was undertaken because the heterogeneity could be accounted for, and a qualitative analysis would still be valuable.

Several studies could not be included in the meta-analysis due to methodological differences. For instance, although Ajami et al. [19] and Koerdt et al. [15] both measured DMFT/dmft ratio scores, they used different approaches to population stratification. Ajami et al. grouped all children aged 3 to 12 years together, while Koerdt et al. divided the population into specific age ranges (3–6 years, 7–12 years, and 13–17 years), preventing direct comparison between the two studies.

Additionally, Frank et al. [20] conducted a retrospective longitudinal cohort study, which introduces time as a factor and is fundamentally different from the cross-sectional design required for this meta-analysis.

Furthermore, Karhumaa et al. [16] reported dt/DT, dmft/DMFT, and DMFT scores for children at various ages (7, 11, and 15 years), but this specific distribution of age groups and indices did not align with the other studies.

Saraç et al. [21] used a totally different method of categorization, dividing participants based on dentition stage (primary, mixed, and permanent dentition) rather than age, which further complicated a direct comparison. While this approach provided valuable insights into the progression of oral health issues across dentition stages, it was incompatible with other studies that grouped participants solely by age.

Some studies, despite offering valuable data, lacked a control group, making them ineligible for meta-analysis but still useful for the broader systematic review. Studies such as Oliver et al. [22], Sethi et al. [23], and Karhumaa et al. [24] provided important insights into the oral health of children with CHD, but without a control group for comparison, their findings could not be quantitatively synthesized.

## 5. Limitations and Perspectives

One of the main challenges in conducting this meta-analysis was the lack of standardization across studies. Although all studies used similar scoring concepts, relying on recording indices of decayed/missed/filled teeth or surfaces in primary and permanent dentition, differences in population grouping, scoring criteria, and study design made direct comparisons difficult.

Moving forward, harmonization in the design and reporting of oral health studies is essential for improving our understanding of oral health outcomes in children with congenital heart disease (CHD). Current studies vary widely in terms of age groups, dentition stages, and scoring indices, which makes it difficult to compare findings or perform comprehensive meta-analyses. For instance, discrepancies arise when studies include or exclude missing teeth in deciduous dentition when calculating caries indices like the DMFT/dmft score.

To address this, future research should adopt more standardized protocols, clearly defining age groups, dentition stages, and the consistent use of caries indices. Such standardization would enhance the comparability of studies, allowing for clearer insights into oral health trends in CHD patients compared to healthy controls. Moreover, using uniform statistical reporting methods and criteria for CHD populations would strengthen cross-study analyses.

By standardizing study designs, future research can facilitate data pooling for more robust statistical analyses and meta-analyses. This would enable stronger evidence-based recommendations for improving oral health care in CHD patients and identifying targeted interventions to reduce their higher risk of dental diseases.

The findings of this systematic review and meta-analysis highlight the importance of early intervention, customized preventive strategies, and collaborative approaches across dental subdisciplines. This underscores the need for coordinated care involving both dental and medical professionals to better address the unique oral health challenges faced by this population.

## 6. Conclusions

This systematic review and meta-analysis revealed that children with CHD tend to have higher dmft and DMFT scores compared to healthy controls, suggesting a potential increased risk of dental caries, particularly as they become older. However, the differences were not uniformly observed across all studies and age groups. Two separate meta-analyses were conducted based on the available data: one analyzing DMFS scores and another focusing on dmft scores. Both meta-analyses showed no significant difference between children with CHD and controls. However, the high heterogeneity in the second meta-analysis emphasizes the need for more standardized study designs and methodologies in the future.

## Figures and Tables

**Table 1:** Search strings.

Database	Search String
Medline	((congenital heart disease[MeSH Terms] OR congenital heart defect[MeSH Terms] OR “CHD” OR “heart malformation” OR “cardiac anomaly”) AND (dental caries[MeSH Terms] OR tooth decay [MeSH Terms] OR caries OR “dental decay” OR “tooth lesions”))
Embase	((“congenital heart disease”/exp OR “congenital heart defect”/exp OR “CHD” OR “heart malformation” OR “cardiac anomaly”) AND (“dental caries”/exp OR “tooth decay”/exp OR “caries” OR “dental decay” OR “tooth lesions”))
Central	((“congenital heart disease” OR “congenital heart defect” OR “CHD” OR “heart malformation” OR “cardiac anomaly”) AND (“dental caries” OR “tooth decay” OR “caries” OR “dental decay” OR “tooth lesions”))

**Table 2:** Characteristics of included studies [7,14,15,16,17,18,19,20,21,22,23,24].

Year	Authors	Title	Study Type	Age	Female	Male	n (CHD)	n (Controls)	Mean CHD	Mean Controls	*p*-Value
2015	Ajami B, Abolfathi G, Mahmoudi E, Mohammadzadeh Z.	Evaluation of Salivary Streptococcus mutans and Dental Caries in Children with Heart Diseases.	Cross-sectional study	3 to 12 years	-	-	50 (CHD), 16 (acquired heart disease)	50	DMFT/dmft (CHD): 2.81 ± 2.74, Acquired heart disease: 1.12 ± 1.25	DMFT/dmft: 3.92 ± 3.71	For 3 groups: 0.013
2018	Oliver KJ, Cheung M, Hallett K, Manton DJ.	Caries experience of children with cardiac conditions attending the Royal Children’s Hospital of Melbourne.	Retrospective study	<12 years	-	-	428	-	dmft: 3.65 ± 4.8, dmfs: 6.19 ± 11.3	-	-
2019	Frank M, Keels MA, Quiñonez R, Roberts M, Divaris K.	Dental Caries Risk Varies Among Subgroups of Children with Special Health Care Needs.	Retrospective longitudinal cohort study	6 months–16 years	-	-	30	30	dmfs (children 6–71 months old): 13.3 ± 18.4, DMFS (>71 months old): 2.4 ± 3.6	dmfs: 0.3 ± 0.9, DMFS: 0.0 ± 0.0	-
2020	Koruyucu M, Batu S, Bayram M, Uslu E, Guven Y, Seymen F	Saliva profiles in children with congenital heart disease.	Cross-sectional study	3–12 years	33.30%	66.70%	42	42	DMFS: 1.02 ± 1.53, dfs: 4.40 ± 3.49	DMFS: 1.14 ± 1.63, Dfs: 5.00 ± 2.94	DMFS: 0.744, dfs: 0.274
2021	Karhumaa H, Lämsä E, Vähänikkilä H, Blomqvist M, Pätilä T, Anttonen V.	Dental caries and attendance to dental care in Finnish children with operated congenital heart disease.	Retrospective study	7, 11, 15 years	44.40%	55.60%	215	3356	dt/DT 7 y: 0.8/0.2 ± 1.6/0.6, dmft/DMFT 7 y: 1.9/0.3 ± 3.2/0.8 dt/DT 11 y: 0.2/0.3 ± 0.7/0.7, dmft/DMFT 11 y: 2.6/0.9 ± 3.6/1.5 DT 15 y: 0.7 ± 1.4, DMFT 15 y: 1.9 ± 2.8	dt/DT 7 y: 0.8/0.2, dmft/DMFT 7 y: –/0.2 dt/DT 11 y: 0.1/0.6, dmft/DMFT 11 y: –/1.3 DT 15 y: 1.2 DMFT 15 y: 2.7	DT 15 y: 0.046 DMFT 15 y: 0.009
2022	Koerdt S, Hartz J, Hollatz S, Heiland M, Neckel N, Ewert P, Oberhoffer R, Deppe H.	Prevalence of dental caries in children with congenital heart disease.	Cross-sectional study	3–17 years	44.90%	55.10%	33 (3–6 y), 63 (7–12 y), 51 (13–17 y)	33, 63, 51	DMFT/dmft (3–6 y): 2.12 ± 1.25, (7–12 y): 2.06 ± 2.27, (13–17 y): 2.12 ± 1.58	DMFT/dmft (3–6 y): 0.99, (7–12 y): 1.26, (13–17 y): 0.93	3–6 y: 0.068, 7–12 y: 0.009, 13–17 y: 0.001
2022	Sethi M, Sood S, Sharma N, Singh A, Sharma P, Kukshal P.	Oral health status and dental anomalies among children with congenital heart disease in contemporary times.	Cross-sectional study	2–16 years	-	-	300	-	DMFT: 0.6 ± 1.6	-	-
2022	Yavsan ZS, Tosun G, Sert A.	Oral health-related quality of life of preschool-aged Turkish children with congenital heart disease.	Cross-sectional study	3–6 years	-	-	75	75	dmft: 5.453 ± 4.366	dmft: 5.360 ± 3.182	dmft: 0.881
2022	Karhumaa H, Vähänikkilä H, Blomqvist M, Pätilä T, Anttonen V.	Behaviour management problems in Finnish children with operated congenital heart disease.	Retrospective study	6, 12, 15 years	-	-	211	-	dt/dmft 6 y: 1.8/9 ± 1.6/3.2, DT/DMFT 6 y: 0.2/0.3 ± 0.6/0.8, dt/dmft 12 y: 0.2/2.6 ± 0.7/3.6, DT/DMFT 12 y: 0.3/0.9 ± 0.7/1.5, DT/DMFT 15 y: 1.7/9 ± 1.4/2.8,	-	-
2023	Bsesa SS, Srour S, Dashash M.	Oral health-related quality of life and oral manifestations of Syrian children with congenital heart disease.	Case-control study	4–12 years	44%	56%	200	100	DMFT: 1.615 ± 1.928 dmft: 5.245 ± 3.982	DMFT: 1.540 ± 1.424 dmft: 2.660 ± 2.244	dmft: <0.05
2023	Saraç F, Derelioğlu SŞ, Şengül F, Laloğlu F, Ceviz N.	The Evaluation of Oral Health Condition and Oral and Dental Care in Children with Congenital Heart Disease.	Descriptive and correlational study	6 months–18 years	48.40%	51.60%	217	364	Primary dentition: dmft 5.4 ± 4 dmfs 9 ± 8.5 Mixed dentition: dft: 5.1 ± 3.2 dfs: 8.2 ± 5.8 DMFT: 3.1 ± 1.7 DMFS: 5.7 ± 5 Permanent dentition: DMFT: 4.1 ± 2.5 DMFS: 7.9 ± 6.1	Primary dentition: dmft: 5 ± 3.4 dmfs: 9.7 ± 9 Mixed dentition: dft: 4.7 ± 2.9 dfs: 10.7 ± 7.4 DMFT: 2.6 ± 1.7 DMFS: 4 ± 3.4 Permanent dentition: DMFT: 4 ± 2.7 DMFS: 6.2 ± 5.5	Primary dentition: dmft: 0.583 dmfs: 0.693 Mixed dentition: dft: 0.532 dfs: 0.047 DMFT: 0.161 DMFS: 0.031 Permanent dentition: DMFT: 0.841 DMFS: 0.151
2024	Hazarika SJ, Jnaneswar A, Jha K.	A Comparative Assessment of Dental Caries Experience in Relation to Nutritional Status among School-going Children with CHD.	Cross-sectional study	6–12 years	48.10%	51.90%	129	257	dmfs: 5.93 ± 10.224, DMFS: 0.33 ± 1.105	dmfs: 3.41 ± 6.192, DMFS: 0.24 ± 0.714	dmfs: 0.031, DMFS: 0.604

**Table 3:** Summary of statistical findings of selected articles.

Study	Age Group (s)	Oral Health Indices	*p*-Value	Outcome
Koerdt et al. [15]	3–6 years	DMFT/dmft	0.068	No statistically significant difference
	7–12 years	DMFT/dmft	0.009	Significant difference, worse oral health in CHD group
	13–17 years	DMFT/dmft	0.001	Significant difference, worse oral health in CHD group
Karhumaa et al. [16]	15 years	DT	0.046	Significant difference, worse DT index in CHD group
	15 years	DMFT	0.009	Significant difference, worse DMFT index in CHD group
Bsesa et al. [14]	4–12 years	dmft	<0.05	Significant difference, higher dmft in CHD group
Hazarika et al. [17]	6–12 years	dmfs	0.031	Significant difference, worse dmfs in CHD group
	6–12 years	DMFS	0.604	No statistically significant difference
Yavsan et al. [7]	3–6 years	dmft	0.881	No statistically significant difference
Koruyucu et al. [18]	3–12 years	DMFS	0.744	No statistically significant difference
	3–12 years	dfs	0.274	No statistically significant difference
	3–12 years	Salivary profile	No *p*-values given	CHD group had lower salivary pH, buffering capacity, and higher oxidative stress markers

## Data Availability

The data presented in this study are available in the article.
